# Receptor for Activated C Kinase1B (RACK1B) Delays Salinity-Induced Senescence in Rice Leaves by Regulating Chlorophyll Degradation

**DOI:** 10.3390/plants12122385

**Published:** 2023-06-20

**Authors:** Md Ahasanur Rahman, Hemayet Ullah

**Affiliations:** Department of Biology, Howard University, Washington, DC 20059, USA

**Keywords:** RACK1B, rice, salt stress, stay-green, light harvesting complex, chlorophyll degradation, senescence, kinases

## Abstract

The widely conserved Receptor for Activated C Kinase1 (RACK1) protein is a WD-40 type scaffold protein that regulates diverse environmental stress signal transduction pathways. *Arabidopsis* RACK1A has been reported to interact with various proteins in salt stress and Light-Harvesting Complex (LHC) pathways. However, the mechanism of how RACK1 contributes to the photosystem and chlorophyll metabolism in stress conditions remains elusive. In this study, using T-DNA-mediated activation tagging transgenic rice (*Oryza sativa* L.) lines, we show that leaves from rice RACK1B gene (OsRACK1B) gain-of-function (RACK1B-OX) plants exhibit the stay-green phenotype under salinity stress. In contrast, leaves from down-regulated OsRACK1B (RACK1B-UX) plants display an accelerated yellowing. qRT-PCR analysis revealed that several genes which encode chlorophyll catabolic enzymes (CCEs) are differentially expressed in both RACK1B-OX and RACK1B-UX rice plants. In addition to CCEs, stay-green (SGR) is a key component that forms the SGR-CCE complex in senescing chloroplasts, and which causes LHCII complex instability. Transcript and protein profiling revealed a significant upregulation of OsSGR in RACK1B-UX plants compared to that in RACK1B-OX rice plants during salt treatment. The results imply that senescence-associated transcription factors (TFs) are altered following altered OsRACK1B expression, indicating a transcriptional reprogramming by OsRACK1B and a novel regulatory mechanism involving the OsRACK1B-OsSGR-TFs complex. Our findings suggest that the ectopic expression of OsRACK1B negatively regulates chlorophyll degradation, leads to a steady level of LHC-II isoform Lhcb1, an essential prerequisite for the state transition of photosynthesis for adaptation, and delays salinity-induced senescence. Taken together, these results provide important insights into the molecular mechanisms of salinity-induced senescence, which can be useful in circumventing the effect of salt on photosynthesis and in reducing the yield penalty of important cereal crops, such as rice, in global climate change conditions.

## 1. Introduction

The Receptor for Activated C Kinase1 (RACK1) is a conserved multifunctional WD-40 type scaffold protein that functions as an assembly platform for diverse signaling pathways that are involved in plant development, stress responses, and immunity [1]. While mammalian RACK1 is well known for its role as a hub for cellular signaling cascades ranging from transcriptional regulation, protein translation, and ribosome machinery biogenesis to cell proliferation, tumorigenesis, immune response, apoptosis, and cell senescence, etc. [2,3,4,5,6], our understanding of the function of plant RACK1 is still emerging. Despite a fundamental difference in phosphorylation sites, plant RACK1s appear to be functionally similar to mammalian RACK1 [7,8]. In plants, RACK1 has been implicated in seed germination, root development, flowering, pollen development, and fruit ripening [9,10,11]. Mutant analysis in *Arabidopsis thaliana* and *Oryza sativa* L. plants revealed that RACK1 plays a crucial role in plants’ response to environmental stress conditions such as drought and salinity [12]. Intriguingly, the regulation of many of these pathways appears to be accomplished through the reported interaction of RACK1 with over 100 different proteins [1,13,14]. However, the physiological role of these interactions remains poorly understood.

The rice genome contains two homologs of the *RACK1* gene—*OsRACK1A* and *OsRACK1B*, with an 82% amino acid similarity. Nakashima et al., in 2008, first reported that OsRACK1A serves as an adapter protein for a complex involving Rac1, RAR1, SGT1, and RbohB at the plasma membrane to provide resistance against blast pathogens [15]. Zhang and colleagues found that OsRACK1A is regulated by the circadian rhythm and interacts with many salt-responsive proteins [12]. In a recent study, it was demonstrated that AtRACK1, the *Arabidopsis* homolog of OsRACK1, participates in leaf senescence [16]. Concurrently, our research revealed that the overexpression of OsRACK1B induces ROS burst as a form of H_2_O_2_ through direct interaction with the N-terminal region of OsRbohD and affects the timing of anther dehiscence, pollen viability, and pollen cell wall integrity [11].

Rice feeds nearly half of the world’s population and is considered to be the single most important source of calories. However, adverse climate change coupled with environmental stressors such as drought, salinity, high and low temperatures, and pathogen attacks significantly constrains rice production. Compared to other cereal crops such as wheat and maize, rice is the most salinity-sensitive crop, and the salinity of the soil causes an annual yield loss of up to 50% in rice-growing countries [17,18,19,20,21]. Salinity affects plants in many ways, including disrupting cellular ionic homeostasis, dismantling the photosynthetic apparatus, breaking down chlorophyll (Chl) pigments, and decreasing the efficiency of photosystem II (PS-II) and PS-I, leading to premature senescence [22,23,24].

Although higher plants undergo senescence processes during the reproductive stage as part of nutrient remobilization for grain and fruit ripening, the process can occur in vegetative stages in harsh environmental conditions. Abiotic and biotic stressors such as salinity, heat, drought, UV radiation, and pathogen attack can induce premature senescence [25,26]. Upon induction by stressors, the highly coordinated process of senescence is set through the activation of transcription factors (TFs) that modulate the expression of senescence-associated genes (SAGs), the breakdown of chloroplast, and the destabilization of the light-harvesting complex, which ultimately leads to cell death [22,27,28,29].

Abiotic stressors such as high salinity promote premature senescence that accompanies the yellowing of leaves due to the degradation of chlorophyll pigments and unmasking of preexisting carotenoids [30,31,32,33]. Chlorophyll retention and delayed senescence, however, lead to the stay-green phenotype, in which leaves remain green in color [34]. Recent research in crop and model plants has demonstrated that a regulatory protein, named stay-green (SGR) or NYE1 (also known as the protein for Mendel’s cotyledon trait), is involved in thylakoid photosystem disassembly and chlorophyll degradation during senescence. Mutant *sgr* plants exhibited a delayed loss of chlorophyll during natural and dark-induced senescence in higher plants [35,36,37,38]. In contrast, SGR- overexpressing plants, specifically rice and *Arabidopsis* seedlings, exhibited reductions in chlorophyll content and accelerated cell death. In *Arabidopsis*, SGR forms a complex with chlorophyll catabolic enzymes (CCEs) for chlorophyll degradation and associates with chlorophyll-binding proteins to aid chloroplast proteases in removing the damaged molecules [33,36,37,39,40]. Agronomically, the stay-green trait and delayed leaf senescence are of great interest for research due to their link with high yields [41]. In conjunction, several recent findings suggest the potential of delayed leaf senescence in grain filling and abiotic stress resilience in cereal crops such as rice and wheat [38,42].

Although RACK1 has been reported to interact with several photosystem complex proteins, to date, there is no report on the precise role that RACK1 plays within the photosystem complex under diverse environmental stimuli. As both RACK1 and the photosystem complex play central roles in plant growth and development, understanding their crosstalk can provide insights into optimizing plant growth and yield potential. With this aim in mind, we sought to investigate RACK1’s role in photosystem complexes under abiotic stress conditions. We used a T-DNA insertional approach, an effective genetic tool that has the potential for the activation (activation-tagging) or suppression of a native transcript depending on the exact position and direction of the insertion site within the genomic sequence [43]. We found that leaf discs from OsRACK1B-overexpressed transgenic rice plants exhibit strong stay-green phenotypes by retaining more chlorophylls than those of the wild-type (WT) plants during salinity-induced senescence. By contrast, leaf discs from down-regulated OsRACK1B plants display chlorotic phenotypes and premature senescence in salinity stress conditions. Gene expression analysis revealed that several genes involved in senescence and chlorophyll catabolism are differentially expressed in the RACK1B transgenic rice plants. Our study also indicates that OsRACK1B plays a key role in protecting the light harvest complex during stress-induced senescence.

## 2. Results

### 2.1. Identification of T-DNA Insertion Activation Tagged Rice Plants Overexpressing and Down-Regulating OsRACK1B

**Figure 1:**
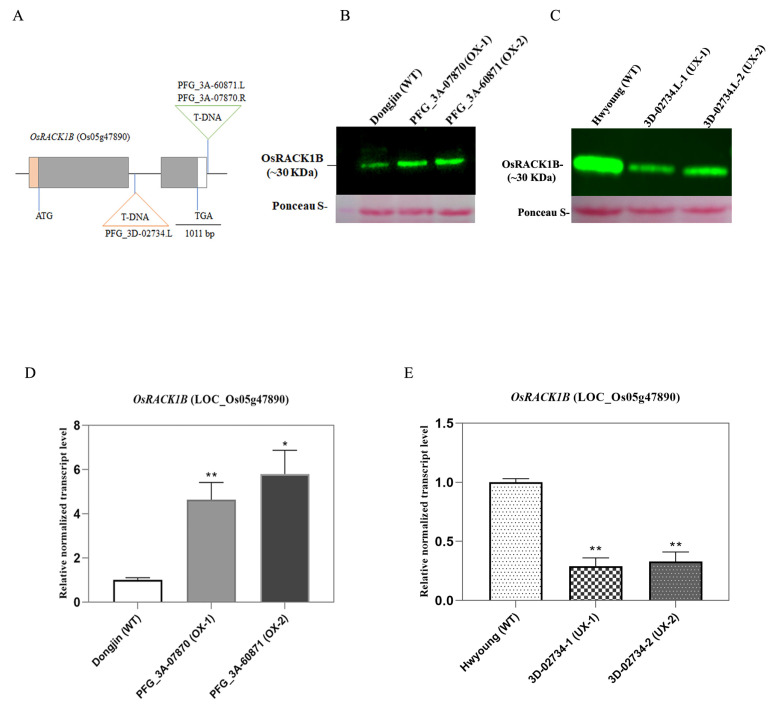
Characterization of T-DNA insertion lines with analysis of gene expression and protein abundance. (**A**) Schematic diagram depicting the positions of T-DNA insertions in *OsRACK1B*. Gray, orange, and white bars represent the exons, 5′-UTR, and 3′-UTR regions, respectively. ATG and TGA are start and stop codons. The gray line represents the intron. The triangles indicate *OsRack1b* mutant alleles (OX-1, PFG_3A-07870.R; OX-2, PFG_3A-60871.L; UX-1 and UX-2, PFG_3D-02734.L). T-DNA insertion in PFG_3A-60871.L and PFG_3A-07870.R lines (green inverted triangle) resulted in RACK1 overexpression shown in B and D. Insertion in PFG_3D-02734.L (orange inverted triangle) is down-regulated allele revealed by expression analysis shown in western blot (**C**) and qRT-PCR (**E**). (**B**,**C**) Western blot analysis of the OsRACK1B in four-week-old wild-type (WT) and *OsRack1b* mutant rice plants. Equal amounts of leaf proteins extracted from the indicated genotypes were subjected to immunoblot analysis stained with anti-OsRACK1B antibody. Ponceau-stained membrane is shown as the loading control. Molecular weight markers are indicated in kDa. (**D**,**E**) qRT-PCR analysis of normalized expression level of *OsRACK1B* in transgenic rice lines compared to their respective wild-type plants. Total RNA was extracted from leaf tissues sampled from detached leaves of four-week-old wild-type and transgenic plants as described in Methods section. *OsActin-1* (LOC4333919) was used to standardize transcript levels in each sample. The data are shown as the means ± SE of three technical repeats. Single (*) and double asterisks (**) indicate significant differences compared with the wild-type values (Student’s *t*-test; * *p* < 0.05; ** *p* < 0.01). Primers are listed in Table S1.

To elucidate the physiological function of OsRACK1B (Loc_Os05g47890) in salt stress, we screened both overexpression and loss of function lines from the RICEGE database (http://signal.salk.edu/cgi-bin/RiceGE, accessed on 16 June 2023). We identified two putative OsRACK1B gain-of-function lines, as described previously in Rahman et al., 2022 [11]. For this study, we identified the PFG_3D-02734.L line as the putative loss-of-function line where T-DNA is located in the intronic region, between the two exons of the same locus. Expression analysis by qRT-PCR and western blot revealed that two plants from putative gain-of-function lines accumulated from four- to seven-fold more *OsRACK1B* transcripts and higher protein levels than those in Dongjin (hereafter termed WT) plants (Figure 1B,D). We refer to these two lines as the OsRACK1B overexpressed lines and designated them as OX-1 (PFG_3A-07870.R) and OX-2 (PFG_3A-60871.L) (Rahman et al., 2022). Similarly, the *OsRACK1B* transcript and protein levels from two plants of the PFG_3D-02734.L line showed a substantial decrease (~50%) compared to the Hwayoung (WT) plant (Figure 1C,E), hereafter referred to as the OsRACK1B knock-down line and designated as UX-1 and UX-2. TAIL-PCR and sequence analysis revealed that the T-DNA insertion sites of both UX-1 and UX-2 are located in the intronic region between the two exons of *OsRACK1B*, 1018 bp downstream of the start codon (ATG) at Loc_Os05g47890 (Figure 1A). Within the nearly 10 kb insertion site, except for several transposons, no other gene coding sequences could be seen, implying a low possibility of interference by the ectopic activation of other nearby genes.

### 2.2. Transgenic Rice Plants Overexpressing OsRACK1B Exhibit Stay-Green Phenotype under Salinity Stress

**Figure 2:**
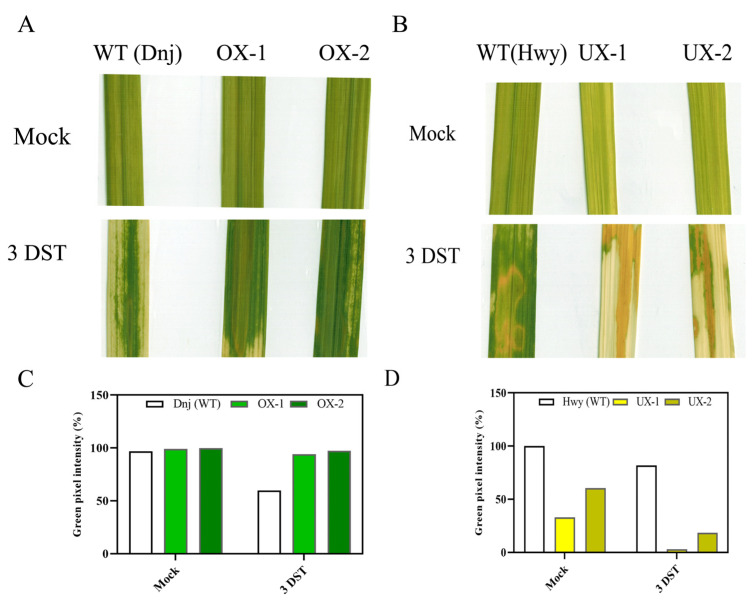
The OsRACK1B-overexpressed (OX) rice leaves retain more chlorophyll than wild-type during high salinity (200 mM NaCl) stress. (**A**) The stay-green phenotype was observed in OsRACK1B-overexpressed OX-1 and OX-2 leaf discs after three days of salt DST (200 mM NaCl) treatment in comparison to the WT leaf discs. (**A**,**C**) Changes in green pigment (measured as the pixel density of the same area by the ImageJ) in leaf discs from eight-week-old OX-1 and OX-2 rice plants after three days of salt treatment (3 DST) and three days of water treatment (Mock) as control. (**B**,**D**), The OsRACK1B-down-regulated (UX) rice leaf discs exhibit rapid yellowing (premature senescence) phenotype than wild-type during salinity stress. (**B**) Expedite chlorotic phenotype was observed in leaf discs from eight-week-old OsRACK1B-down-regulated UX-1 and UX-2 leaf discs after three days of salt (200 mM NaCl) treatment in comparison to the WT. (**D**) Green pigment changes (measured as the pixel density of the same area by the ImageJ) in leaves of UX-1 and UX-2 plants after three days of salt treatment (3 DST) and three days of water treatment (Mock) as control.

Despite having molecular evidence of RACK1 being an important regulator in response to salt stress, there has been a significant gap in understanding regarding the physiological role of RACK1 in stress-induced metabolic pathways, such as premature senescence. It was of interest to test whether RACK1 also contributes to this process concomitantly with salt-stress signaling pathways. To this end, we investigated the physiological function of RACK1B in response to high salinity-induced senescence. Previous studies suggest that treatment with high salinity such as 200 mM NaCl for several days is one of the approaches to understanding the initiation, progression, and molecular mechanisms of senescence [44,45,46]. As such, the leaf discs from eight-week-old OX-1, OX-2, and WT plants were subjected to 200 mM salt stress for three days under continuous light conditions. After three days of salt treatment (DST), the phenotyping analysis revealed that leaf discs from the OX-1 and OX-2 plants exhibited stay-green phenotypes, while those of the WT turned yellow, suggesting a delayed chlorophyll degradation in the OX plants (Figure 2A,C). The ImageJ analysis of a similar area size revealed that chlorophyll pigmentation was higher in the OX leaves than in the WT leaf discs. Consistent with the visible phenotype, the OX leaf discs retained more total chlorophyll than the WT leaf discs after three days of salt treatment (Supplementary Figure S3A).

### 2.3. OsRACK1B Down-Regulated Plant Leaves Display Premature Senescence in Salinity Stress

To further confirm the effect of RACK1 in chlorophyll catabolism under salinity stress conditions, we subjected leaf discs from down-regulated RACK1B plants (UX-1 and UX-2) to 200 mM NaCl for three days under continuous light conditions. After three days, the UX leaf discs exhibited severe yellowing compared to the WT leaf discs, which remained partially green (Figure 2B). In accordance with visible phenotypes, the ImageJ analysis of similar areas revealed that chlorophyll pigmentation was also lower than WT leaf discs (Figure 2D). The yellow phenotype in UX rice leaf discs indicated expedited chlorophyll degradation and early senescence compared to that in the WT leaves during salt stress. This phenotype contrasts with that of the OsRACK1B-OX leaves treated with the same concentration of salt for three days. Please note that the WT controls are different for the OX and UX lines. The total chlorophyll quantification also reflected the visible phenotype, as UX rice leaves contained a significantly low amount of chlorophyll compared the WT after three days of salt treatment (Supplementary Figure S3B). To exclude the possibility of external factors influencing this phenotype, the plants were grown and leaf discs were treated under the same conditions. The phenotypic differences in the OsRACK1B-OX and UX leaf discs demonstrated that RACK1B is closely associated with salinity-induced senescence. To this end, we propose that RACK1B may act as a negative regulator of stress-induced chlorophyll degradation in rice plants. To rule out other possibilities influencing the phenotype, we screened the selected OsRACK1B transgenic lines for additional insertions in different chromosomal locations using Thermal Asymmetric Interlaced PCR (TAIL-PCR), as described in Rahman et al., 2022 [11]. While OX-1, OX-2, and UX-1 plants were confirmed to be a single copy of T-DNA insertion, a second insertion was found (Supplementary Figure S1A,B) near an EF-hand domain-containing protein (Loc_Os08g0558100) at Chr8 in the UX-2 plant (Supplementary Figure S2A,B) and, consequently, was discarded from further investigation.

### 2.4. OsRACK1B Negatively Regulates the Expression of Chlorophyll Degradation and Senescence-Associated Genes

**Figure 3:**
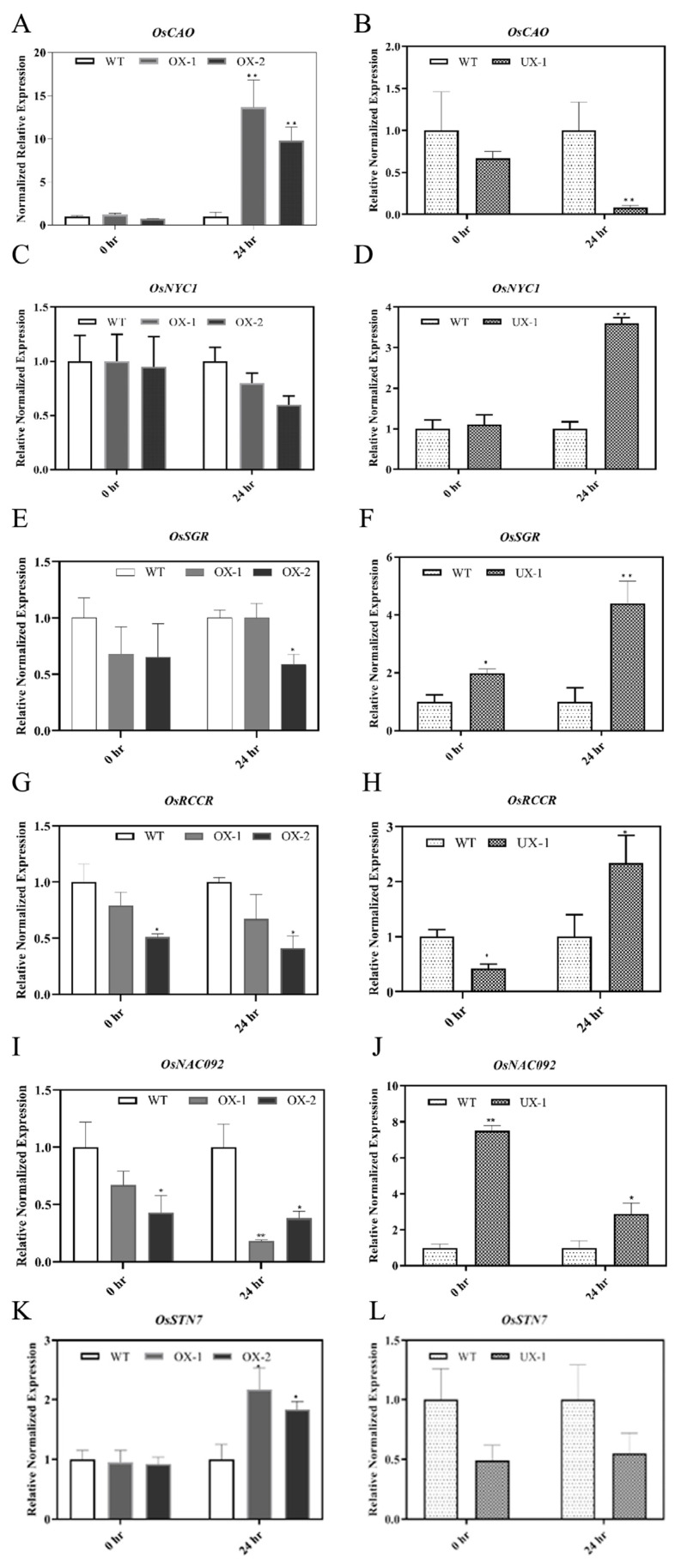
Altered expression of chlorophyll metabolic genes in leaves of OsRACK1B OX and UX plants under salinity stress condition. The transcript levels of chlorophyll biosynthetic gene chlorophyllide a oxygenase, *CAO* (**A**,**B**); chlorophyll catabolic enzyme encoding genes NONYELLOW COLORING1, *NYC1* (**C**,**D**), and red chlorophyll catabolic reductase, *RCCR* (**G**,**H**); chlorophyll degradation related gene, Stay-green *SGR* (**E**,**F**); senescence-associated transcription factor, *NAC092* (**I**,**J**); and LHCII phosphorylation and state transition related gene Serine/threonine protein kinase, *STN7* (**K**,**L**) were analyzed using qRT-PCR. Total mRNA was extracted from leaf tissue from eight-week-old OsRACK1B-overexpressed (OX-1 and OX-2) and OsRACK1B-down-regulated (UX-1) plants, and their respective wild-type (WT) plants (Dongjin and Hwayoung), at 0 h and after 24 h of salt (200 mM NaCl) treatment. (**A**–**L**) The transcript levels of all genes were normalized using OsActin-1 (LOC4333919). Error bars indicate the standard errors (SE) of the means (*n* = 3) and values are means ± SE. Single and double asterisks indicate statistical significance: * *p* < 0.05 and ** *p* < 0.01 compared to the wild-type (Student’s *t*-test). Primers are listed in Table S2.

Leaf yellowing is the primary symptom of senescence and can be initiated by aging and environmental elicitors such as high salinity. In higher plants, senescence induction is highly associated with chlorophyll metabolism [31,47]. Several genes involved in chlorophyll synthesis or degradation are differentially expressed during senescence [47,48]. To elucidate the molecular mechanism of how the overexpression or down-regulation of RACK1 perturbs the chlorophyll metabolism, we used quantitative PCR (qRT-PCR) to measure the transcript levels of some key genes involved in chlorophyll metabolism at baseline (0 h) and after 24 h of salt treatment. First, we measured the expression of a chlorophyll biosynthesis gene: *chlorophyllide a oxygenase (CAO)*, which encodes Chl a oxygenase that catalyzes the transformation of Chl a into Chl b [49]. Gene expression analysis revealed that *OsCAO* levels are significantly up-regulated in OX plants and down-regulated in UX plants in comparison to WT levels following salt treatment (Figure 3A,B). Previously, it was found that plants overexpressing CAO exhibit the stay-green phenotype and that several transcription factors were differentially expressed when artificial senescence was induced [50]. Therefore, we hypothesized that OsCAO upregulation in OX plants after salt treatment is one of the reasons for its stay-green phenotype in leaves upon salt exposure. Likewise, consistent with chlorotic phenotypes, low *OsCAO* expression in the UX plant suggests rapid chlorophyll breakdown in UX plant under high salinity. Next, we measured the mRNA expression of key genes which encode chlorophyll catabolic enzymes (CCE). Chlorophyll degradation is a multi-step process. The cascade starts with the conversion of Chl b to Chl a, catalyzed by Chl b reductase (NYC1). In the final step, the ring structure of the intermediate breakdown products is oxygenolytically opened by pheophorbide a oxygenase (PAO) to generate red chl-catabolite (RCC), which is degraded further by RCC reductase (RCCR). Several studies in plants found that mutations of NYC1 and PAO lead to chlorophyll retention, and their transcript levels increase during natural or salinity-induced senescence [31,34,51,52]. We found that the transcript levels of *OsNYC* and *OsRCCR* were up-regulated in the UX plant after salt exposure in comparison to that in the WT plant (Figure 3D,H). By contrast, their expression pattern in OX plants remains the same or even lower after 24 h of salt treatment (Figure 3C,G). Therefore, we speculated that the constitutive expression of OsRACK1B negatively influences the expression of CCEs, which is consistent with their stay-green phenotype in OX plants. Likewise, accelerated leaf yellowing in UX plant indicates that functional RACK1 plays an important role in limiting the expression of CCE genes. Senescence is a complex process that is tightly controlled by several senescence-related transcriptional factors (TFs). We questioned whether RACK1B overexpression and down-regulation have any effect on transcriptional reprogramming during salt stress-induced senescence. In plants, NAC (NAM, ATAF1, -2, and CUC2) domain transcription factors represent the largest family members in transcriptional regulation and are well-known for their critical roles in stress-induced senescence [47]. One of the TFs—OsNAC092 (also called ORE1)—has recently been identified as a key transcriptional activator during abiotic stress-dependent senescence. *OsNAC092* gene expression is triggered by salt stress and activates a large number of senescence-associated genes [28,53]. As anticipated, we found a significant (up to seven-fold) up-regulation of *OsNAC092* in the UX plant compared to that in the WT plant, indicating a possible activation of a large number of SAGs (Figure 3J). By contrast, its expression was significantly lower in the OX plants (Figure 3I) after salt treatment, and this level was in good accordance with their stay-green phenotype.

### 2.5. OsRACK1B Regulates the Expression of OsSGR

**Figure 4:**
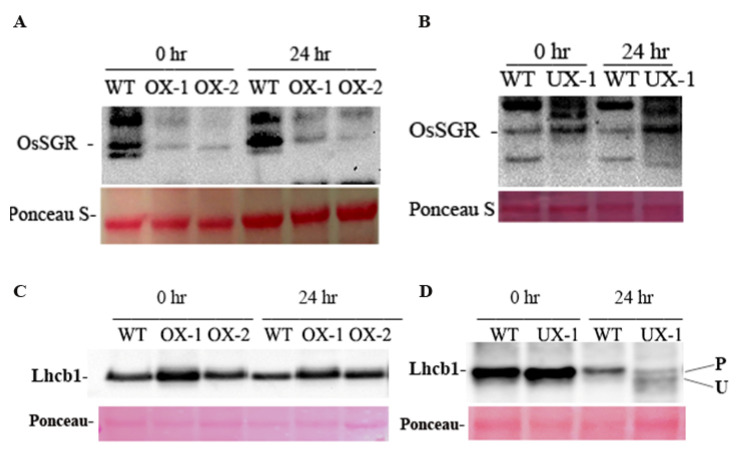
Immunoblot analysis of Photosynthetic Proteins. The altered level of OsRACK1B protein causes a change in the abundance of Lhcb1 and stay-green protein (SGR1) during salinity-induced leaf senescence. Total protein was extracted from harvested leaves from eight-week-old transgenic rice plants and their respective WT plants at 0 h and 24 h of salt (200 mM NaCl) treatment. Anti-Lhcb1 and anti-SGR antibodies were used for immunoblot analysis. (**A**,**B**) Immunodetection of OsSGR. (**C**,**D**) Immunodetection of OsLhcb1. Antisera against Lhcb1 recognized two bands in the UX-1 sample. Upper band (slower migrating band) represents the phosphorylated form. P and U indicate the phosphorylated and unphosphorylated forms, respectively. Each lane contained equal amount of total protein from leaf tissue.

Mendel’s green cotyledon gene stay-green (SGR), that encodes Mg-dechelatase, has recently been recognized as a central regulator of natural and stress-induced chlorophyll breakdown [35,38,54,55,56,57]. SGR interacts with all known CCEs and forms a multiprotein complex with CCEs and LHCII proteins for chlorophyll degradation, the proteolytic cleavage of chloroplast, and the channeling of phototoxic intermediate materials into vacuoles [36,37,58,59]. We speculated that RACK1B may regulate SGR, and this regulation might be associated with chlorophyll catabolism in transgenic rice plants. In agreement with the observed phenotype, transcript and protein profiling revealed that the SGR level is significantly altered in transgenic plants, especially after salt stress. qRT-PCR analysis revealed that *OsSGR* mRNA was much more abundant in the UX plant before and after salt treatment than that in the WT plant; conversely, the expression remains lower in OX plants (Figure 3E,F). To confirm that OsSGR is indeed a target of translational regulation, we analyzed OsSGR protein expression with western blot analysis. When protein extracts before and after salt treatment from the leaves of the OX, UX, and respective WT plants were gel electrophoresed and stained with an anti-SGR antibody, we found differences in the SGR protein levels between the OX and the UX plants compared with that in the WT. Consistent with their mRNA level, the OX/SGR protein level was lower compared to that in the WT/SGR, indicating a down-regulation of SGR protein (Figure 4A) compared to their respective untreated WT cells, possibly due to constitutive expression of RACK1B. Even after 24 h of salt treatment, the SGR protein level was significantly high in the WT cells, but the level remained steady in the OX cells. Similarly, the down-regulation of RACK1B increased the SGR level, as the UX/SGR band showed high intensity compared to the WT/SGR band (Figure 4B). High salinity further induced the SGR expression in the UX plant compared with that in the WT plant (Figure 4B). Collectively, these findings provided compelling evidence that SGR is regulated at both the transcriptional and translational stage by RACK1B during natural and high salinity conditions.

To test whether OsSGR is regulated by OsRACK1B through direct interaction in plants, we performed bimolecular fluorescence complementation (BiFC) assays (Supplementary Figure S4A). Microscopic examination revealed a strong reconstituted YFP fluorescence when nYFP-RACK1B is co-expressed with OsSGR-cYFP (Supplementary Figure S4B). The yellow fluorescent signal co-localized with the blue fluorescence of DAPI, thus confirming an interaction between OsRACK1B and OsSGR in the nucleus (Supplementary Figure S4B, top panel). Similarly, the fluorescence could be seen in both the nucleus and cytoplasm when onion peels were treated with 200 mM NaCl for 30 min (Supplementary Figure S4B; second panel). This result implies that OsRACK1B negatively regulates chlorophyll degradation during salt-induced senescence at least in part by directly interacting with OsSGR.

### 2.6. A Functional OsRACK1 Is Required for LHCII State Transition

Previously, it was shown that AtRACK1 interacts with chlorophyll a/b binding proteins in the light-harvesting complex II (LHCII) [13]. As SGR interacts with all known CCEs [54,60,61] in the LHCII during chlorophyll breakdown, we investigated whether the physical interaction between RACK1B and SGR would have an effect on the PSII-LHCII super-complex and on PSII stability, especially under stress conditions. To gain further insight into the effect on PSII-LHCII, a western blot analysis of light-harvesting chlorophyll a/b binding protein 1 (Lhcb1), one of the two major LHCII proteins, was performed. Lhcb1 is the most abundant in the LHCII super-complex among the trimers Lhcb1, Lhcb2, and Lhcb3 [62]. It undergoes rapid phosphorylation that is catalyzed by a protein kinase-STN7 and dephosphorylation during the state transition from PSII to PSI [62,63,64]. The process of state transition is one of the mechanisms adopted by plants to tune the performances of PSI and PSII, especially during stress adaptation [65,66]. To investigate whether the Lhcb1 expression level is perturbed in RACK1B transgenic lines, we performed a western blot analysis. We found that the expression of Lhcb1 in the OX and UX plants remained similar, as compared to their respective WT plants in normal conditions. However, after 24 h of salt treatment, the level remained stable in the OX plants but slightly decreased in the WT plant (Figure 4C). By contrast, the Lhcb1 protein level in the UX-1 plant had been reduced significantly compared to that in the WT plant after 24 h of salt treatment (Figure 4D). Furthermore, we observed an accumulation of an apparent non-phosphorylated form of Lhcb1 in the UX plant after salt treatment, whereas it was completely absent in the OX plants and their respective WT plants (Figure 4C,D). These observations suggest that the loss of function of RACK1B led to the loss of LHC integrity, which is an essential prerequisite for the state I-to-state II transition and triggers the dissociation of LHCII from PSII to PSI. It also suggests the possibility of an activated STN7 in OX plants during salt treatment. Indeed, the qPCR analysis revealed an increased mRNA level of *STN7* in the OX plants after salt treatment (Figure 3K). Similarly, a reduction in phosphorylated Lhcb1 in the UX plant indicates that the LHC failed to maintain a smooth transition between PSI and PSII in the absence of a functional RACK1. This is also evidenced by the appearance of an apparent non-phosphorylated Lhcb1 in the UX plants. It is, therefore, likely that the degradation of LHC is due to increased activity of SGR and/or low activity of STN7, particularly under salt stress conditions. Notably, SGR was found to be coimmunoprecipitated with Lhcb1 [33,67]. Interestingly, the low mRNA expression level of STN7 (Figure 3L) also agrees with the findings of the increased non-phosphorylated band in the UX plant.

## 3. Discussion

In plants, the regulatory network of RACK1 has been well-studied in the model plant *Arabidopsis*, but little is known for monocot species, such as rice, as there have been only a few reports made available to date. Kundu et al. (2013) [13] used AtRACK1A as bait to screen a split-ubiquitin-based inflorescence cDNA library and demonstrated that AtRACK1A interacts with a significant number of the proteins found in photosynthesis and the light-regulated physiological processes in normal and stress conditions. OsRACK1 was reported to be regulated by circadian rhythms and to be involved in the regulation of salt stress responses, and was also found to be colocalized with PsbP, an extrinsic subunit of photosystem II (PSII) [12]. However, the regulatory network of RACK1’s role in photosynthesis-related processes remains unclear. To further dissect the role of RACK1 in the photosystem complex and salt response, we investigated the physiological and molecular response of a monocot model and economically important crop, the rice plant, to salinity stress. Here, we have provided several lines of evidence which show a novel function of OsRACK1B in regulating chlorophyll metabolism and salinity-induced senescence. We show that OsRACK1B-OX leaves retain chlorophyll by inhibiting the functional expression of key chlorophyll catabolic genes (CCGs), namely *SGR*, *NYC1*, *RCCR*, and *PAO*, under senescence-promoting high salinity conditions. Our study revealed that leaves from OsRACK1B-OX plants exhibit persistent greenness or the “stay-green” phenotype much longer than WT leaves during high salt stress conditions (200 mM NaCl). By contrast, under the same conditions, OsRACK1B-UX leaves display more leaf yellowing, an early senescence phenotype, than the WT leaves. Therefore, we speculated that the stay-green phenotype in OsRACK1B-OX plants or premature senescence in OsRACK1B-UX plants under stress conditions is due to the initiation of the cellular programs for chlorophyll catabolism. Indeed, this assumption is supported by the findings that, in the OsRACK1B-OX plants, chlorophyll catabolic genes were down-regulated. Conversely, more enhanced levels of chlorophyll catabolic gene expression were observed in the RACK1B-UX plant than in the WT plant. These results indicate that OsRACK1B plays a critical role in stress-induced leaf senescence. Notably, using RNA-interfered transgenic rice plants, Zhang et al., 2018, found that the suppression of OsRACK1A increased the chlorophyll content under 150 mM NaCl stress for up to 72 h [12]. This disagreement may be attributed to the differences in the amino acid sequence and expression pattern depending on the tissue-specific location of the two homologs. Although OsRACK1A and OsRACK1B share 82% of their amino acid identity, a distinct function for each of the genes is evident from discernable phenotypes from single-gene manipulations. In fact, using double and triple mutants of *RACK1* genes in *Arabidopsis*, it was shown that the genes act with unequal redundancy [10]. Previously, our lab reported that, in addition to key residue phosphorylation-based interactions with more than 100 different proteins, the RACK1 proteins homo- and hetero-dimerize [68]. In this context, an epistatic relationship between these two RACK1 proteins in the chlorophyll catabolism pathway is quite possible. The chlorophyll catabolic pathway in plants has been intensively studied and most, if not all, of the genes involved are isolated and known as CCEs. In rice, some key genes such as *OsSGR*, *OsNYC1*, and *OsRCCR* have been recognized as hallmark genes for leaf senescence [34,36,69,70,71]. Our results show that, under strong abiotic stress conditions, RACK1B-OX leaves maintain more chlorophylls and RACK1B-UX leaves turn yellow much faster than WT leaves. Thus, regulation of the expression of CCE genes in these transgenic plants was not surprising. Indeed, when eight-week-old plants are treated with 200mM NaCl for 24 h, we found low expressions of *OsSGR*, *OsNYC1*, and *OsRCCR* genes in the RACK1B-OX plants. In contrast, the expression of these genes is significantly induced in the RACK1B-UX plant. This implies a substrate delimited catabolic feedback pathway where the limited availability of substrate molecules would impede the downstream catabolic activities—hence a down-regulation of the catabolic enzymes in a sequential pathway. Under abiotic stress conditions, chloroplast homeostasis is maintained through fine coordination with nuclear gene expression for coping with stress. Intriguingly, although all CCEs are located in the chloroplast, a majority of the chloroplast proteome is nucleus-encoded and must be translocated to plastids after their synthesis in the cytosol [33,34,72,73,74]. Therefore, the regulation of nuclear gene expression in response to the functional or metabolic state of the plastids, driven by retrograde signals from the plastids, is essential [75]. In rice, RACK1 was found in the nucleus, cytosol, and microsomal fractions [15,76]. RACK1 was also found to shuttle proteins around the cell [11,77,78]. Evidence suggests that RACK1′s ability of the temporal and spatial regulation of diverse signal transduction can be attributed to its translocation among various cellular compartments, which is influenced by the particular cohort of proteins interacting with RACK1 at any given time [2,9]. Moreover, the lack of specific localization motifs in RACK1′s sequence also contributes to this ability. Thus, it is assumed that RACK1B itself regulates transcription factors in the nucleus or facilitates the translocation of components for transcriptional re-programming as an adaptor protein. For instance, RACK1A interacts with the Osrap2.6 transcription factor in the cytoplasm and nucleus, and contributes to the innate immunity of rice [76]. Conversely, RACK1A also interacts with Rac1 at the periphery of the plasma membrane to exert the same function [15]. Whether RACK1 has any role in chloroplast ribosome biogenesis is still unknown. Remarkably, chloroplast ribosome-associated proteins are found to support translation during stress conditions [79]. Over the last decade, several functional studies in *Arabidopsis*, rice, and other plants have implicated SGR as one of the master regulators of Chl degradation, and *sgr* mutant plants displayed delayed the Chl-degradation phenotype during natural and stress-induced senescence [55,56,69,80,81,82,83]. Rice has two SGR homologs, SGR and SGR-like (SGRL), with overlapping biochemical functions [59]. The *Arabidopsis* genome contains three homologs—*SGR1*, *SGR2*, and *SGRL* [37]. In rice, the overexpression of both OsSGR and OsSGRL causes leaf yellowing during stress-induced and natural senescence processes [35,40,59]. In contrast, *ossgr* rice plants exhibit a strong stay-green phenotype [36,84]. Intriguingly, in *Arabidopsis*, SGR1 and SGRL positively regulate Chl degradation, while SGR2 is a negative regulator of Chl catabolism during natural and stress-induced senescence, despite the 76% amino acid sequence similarity between SGR1 and SGR2 [38,45,51,67]. Our experiment revealed that SGR expression is significantly induced in RACK1-UX leaves, while its expression is considerably reduced in RACK1B-OX leaves, even with 200 mM NaCl treatment for 24 h. These findings indicated that the altered transcript and proteomic expression of OsSGR directly correlates with the altered expression of OsRACK1B in both normal and high salinity conditions. SGR interacts with six chlorophyll degradation enzymes (CCEs), including NYC1 and RCCR [33]. Taken together, based on the expression patterns of OsSGR and CCEs in our experiment, we assumed that OsRACK1B most likely inhibits the function of OsSGR by physically interacting with it, especially under stress conditions. We tested this hypothesis by using BiFC analysis in onion epidermal cells. Our analysis revealed that OsRACK1B interacts with OsSGR in the nucleus and outside of the nucleus under normal and high salinity stress conditions, suggesting a transcriptional and post-transcriptional regulation of OsSGR by OsRACK1B in chlorophyll catabolism. However, the use of green tissues for such activities would have helped to evaluate whether the interaction in the chloroplast is a pre-requisite for inhibiting SGR activities, which functionally take place within the chloroplast. Although SGR localizes to the thylakoid membrane of chloroplasts [33], reports suggest that SGRs do not only occur in chloroplasts. Its functional presence and interaction with other proteins were also observed in different locations [82,85]. Elucidating the detailed mechanisms of how OsRACK1B bonding with OsSGR inactivates SGR activity or represses its expression requires further studies. Two mechanisms can be postulated for how RACK1B prevents chlorophyll degradation by regulating OsSGR: the most likely possibility is that, by forming a heterodimer with SGR, RACK1B limits the availability of SGR to form the SGR-CCE-LHCII multiprotein complex, a prerequisite for chlorophyll breakdown [38]. This hypothesis can be substantiated by recent findings which show that, in *Arabidopsis*, SGR2 negatively regulates chlorophyll degradation by forming homo- or hetero-dimers with SGR1 [38,45,67]. Considering that SGR2 itself can bind to LHCII, the authors proposed that the hetero-dimerization of SGR1 with SGR2 interrupts the formation of the SGR1–CCE–LHCII protein complexes, and thereby limits Chl degradation under stress conditions.

Since there is no report of an orthologue of SGR2 in rice, the question of whether rice SGRL functions similarly through the formation of a heterodimer with SGR needs further investigation. Another possibility is that tethering between RACK1B and SGR triggers a conformational change that makes the complex a better substrate for proteases. A wide range of chloroplast proteins undergo degradation and removal processes such as autophagy, senescence-associated vacuole (SAV) lysis, and intra-plastidial proteolysis by different forms of proteases (such as Clp, FtsH, etc.) for the biogenesis and maintenance of chloroplasts, especially under stress conditions [29,86,87,88]. Of interest, a similar finding was observed in *Arabidopsis*: chloroplast vesiculation (CV) protein interacts with photosystem II subunit PsbO1 via a highly conserved C-terminal domain and appears to alter the structure and stability of the PSII complex, consequently facilitating the degradation of core proteins such as D1 by thylakoid proteases under salt stress conditions [89]. We do not know yet whether OsSGR is subject to such recognition of different N or C termini, modified or not, by the proteases in the chloroplast. Further studies are required to address this potentially important link. Previous studies have demonstrated that, in rice and *Arabidopsis*, SGR forms a complex together with CCEs and LHCII proteins such as Lhcb1, but not with LHCI or photosystem core proteins. The phosphorylation of LHCII is required for the transition from state-I to state-II for adaptation in response to environmental changes [65,90]. A serine-threonine protein kinase named STN7 is necessary for phosphorylation of the LHCII to regulate the reversible association between PSII and LHCII during PSII repair [63,91,92]. Our expression analysis revealed a steady level of light-harvesting chlorophyll a/b binding 1 (Lhcb1) protein RACK1B-OX plants, suggesting the maintained integrity of the LHCII complex in response to salinity stress. Conversely, the high activity of SGR prompted the degradation of the LHCII complex, as reflected in the low amount of Lhcb1 protein and reduced expression of *STN7* coinciding with the amount of non-phosphorylated Lhcb1. Since state transitions depend on STN7 kinase [63], our data can be linked to the functional stability of the chloroplast and a redox balance between PSII and PSI for photosynthetic efficiency in response to stress conditions. Being localized mostly in the nucleus and cytoplasm, it is unclear whether RACK1 shuttles photosynthetic proteins in the chloroplast to form a large complex in the photosynthesis process or works as a molecular chaperone for structural remodeling of the light-harvesting complex during state transitions. Given that RACK1 interacts with chlorophyll a/b binding protein, we cannot rule out the possibility that OsRACK1B might interact with other CCEs or LHCII subunits. Taken together, we conclude that RACK1B is required for the dynamics of PSII–LHCII supramolecular complexes under stress conditions. There are limitations to our study. First, we could not confirm whether the stay-green phenotype is functional or cosmetic stay-green or whether leaves of OsRACKB-OX plants were active in photosynthesis during salinity stress condition. This is important, since functional stay-greens retain both Chl and photosynthetic capacity longer than the WT, and cosmetic stay-greens retain Chl but undergo other aspects of senescence similar to WT [41]. This can be assessed in the future using Fv/Fm measurements. Second, we could not test if the SGR and RACK1B proteins coexist in a similar multiprotein protein complex. A pull-down assay would confirm the hypothesis. Also, our present experimental approach of using BiFC in onion peel cells did not allow us to evaluate whether the cellular localization of RACK1B-SGR interaction has any influence on chlorophyll catabolism. Since SGR localizes to the thylakoid membrane of chloroplasts and carries out chlorophyll breakdown by forming the SGR-CCE-LHCII multiprotein complex, further studies are needed to prove the interaction between RACK1B and SGR in plant cells with chloroplasts such as *Arabidopsis* mesophyll cell protoplasts.

## 4. Materials and Methods

### 4.1. Plant Materials, Growth Condition, and Stress Treatment

We identified OsRACK1B T-DNA insertion lines from the Rice functional genomic express database (http://signal.salk.edu/cgi-bin/RiceGE, accessed on 16 June 2023). T-DNA tagged lines PFG_3A-60871.L and PFG_3A-07870.R, both in the Dongjin (*Oryza sativa* ssp. *Japonica* cv. Dongjin) background and PFG_3D-02734.L in the Hwayoung (*Oryza sativa* ssp. *Japonica* cv. Hwayoung) background seeds, were purchased from Crop Biotech Inst., Korea [93,94]. Seeds were surface sterilized and germinated on full strength Murashige and Skoog (MS) [95] culture media (Caisson Laboratories, Inc., Smithfield, UT, USA) at room temperature. Leaves from two-week-old germinated seedlings were used for DNA extraction and genotyping. Wild-type (WT) and selected transgenic lines were then transferred to hydroponic nutrient solution prepared following the protocol as described by Lakshmanan et al., 2015. Plants were grown at 28 °C during a 14 h light (300 μmol m^−2^ s^−1^) period and 24 °C during 10 h of darkness with 60% relative humidity.

For salt stress treatment, eight-week-old plants were transferred to hydroponic solution in which NaCl (200 mM) was added gradually. After 24 h, plants were washed with tap water and placed in a fresh hydroponic solution for recovery. Leaf tissues from plants were sampled before and after salt stress, flash-frozen in liquid nitrogen, and stored at –80 °C for protein and RNA extraction.

### 4.2. Genotyping of the T-DNA Flanking Region of OsRACK1B Transgenic Lines

Transgenic lines were screened for possible T-DNA insertions positioned near RACK1B (Loc_Os05g47890) by PCR. Genotyping of RACK1B overexpression lines was performed according to the protocol described in Rahman et al., 2022 [11]. For putative loss of function lines, similar genotyping PCR was performed for Salk line PFG_3D-027334 using T-DNA and gene-specific primers, revealing T-DNA insertion in two rice plants from PFG_3D-02734 lines (Supplementary Figure S1).

### 4.3. RNA Extraction, Complementary DNA (cDNA) Synthesis and Quantitative Reverse Transcriptase PCR (qRT-PCR) Analysis

Total RNA was extracted before and after salt stress from eight-week-old rice leaves. Freshly harvested leaves were ground in fine powder using liquid nitrogen and pre-chilled mortar and pestle. A total of 100 mg of powdered tissue sample was used for RNA extraction using the RNeasy Plant Mini Kit (Qiagen, MD, USA). Finally, 500 ng of total RNA for each sample was reverse transcribed to make cDNA using SuperScript IV VILO Master Mix kit (Thermo Fisher Scientific, Waltham, MA, USA) following manufacturer’s instruction.

Quantitative Reverse-Transcriptase PCR (qRT-PCR) was performed using cDNA and PowerUP SYBR Green master mix (Thermo Fisher Scientific, MA, USA) with a CFX96 real-time PCR detection system (Bio-Rad, Hercules, CA, USA). Normalized expression (DDC(t) method) was calculated using the Bio-Rad CFX manager software, employing the housekeeping gene *OsActin1* (Os03g50885) as a reference gene. All experiments were performed in triplicate for technical repeats. The results were plotted as relative values ±SEM and graphically displayed using GraphPad Prism version 8.2.0 (GraphPad Software Inc., Boston, MA, USA). The primer sequences used are listed in Supplementary Table S2.

### 4.4. Protein Extraction and Western Blot Analysis

Total protein from leaves was extracted from 100 mg of finely powdered tissue using lysis buffer (CelLytic P, Sigma-Aldrich, St. Louis, MO, USA) containing protease and phosphatase inhibitor cocktail (Sigma-Aldrich, MO, USA). Total protein content for each sample was quantified by Bradford assay using Quick Start Bradford dye reagent (Bio-Rad, CA, USA).

Western blot analysis was performed using an equal amount of total proteins per sample and resolved in 4–12% pre-cast XT-MES gel (Bio-Rad, CA, USA). The proteins were transferred to polyvinylidene difluoride (PVDF) membrane, and incubated with primary polyclonal antibodies and secondary antibodies. Membranes were developed using the ECL detection kit (Bio-Rad, CA, USA). The signals of the bands were visualized and captured using the Chemi-DocXRS system (Bio-Rad, CA, USA). The OsRACK1B peptide-specific mAb antibodies were raised using two peptides—AGVLRGHNDM and QDLKPEVQAF—corresponding to amino acids 10–19 at the N-terminus and 286–295 at the C-terminus end OsRACK1B protein sequences, respectively (Abmart, Shanghai, China). Commercially purchased Anti-Lhcb1 (cat# AS01004 Agrisera, Vännäs, Sweden) antibody used in the study demonstrated reactivity as stated on the manufacturer’s website and as shown previously by Liu et al., 2019. Similarly, a polyclonal Anti-SGR (Cat# PHY1024S, PhytoAB Inc., San Jose, CA, USA) antibody was used. Because of the high conservation and specificity of the peptides, anti-SGR antibody derived from synthetic peptide using *Arabidopsis* SGR (AT4G22920) was used to detect OsSGR. Antibody binding was validated against *Arabidopsis* and rice protein samples to confirm reactivity and specificity against SGR protein (data not shown). Ponceau-S (40% methanol (*v/v*), 15% acetic acid (*v/v*), and 0.25% Ponceau-S) staining of Rubisco was used as loading control.

### 4.5. Bimolecular Fluorescence Complementation (BiFC) Assay

For BiFC assay, total RNA was extracted from eight-week-old wild-type rice leaves using Trizol reagent (Thermo Fisher, CA, USA) according to the manufacturer’s instructions. A total of 1 µg of purified total RNA per sample was reverse transcribed using SuperScript™ IV VILO Master Mix kit (Thermo Fisher, CA, USA) following manufacturer’s instruction. Full-length coding sequences of OsRACK1B and OsSGR with or without stop codons were amplified by PCR using Q5^®^ High-Fidelity DNA Polymerase (NEB, Ipswich, MA, USA). Amplicons were purified (QIAquick PCR Purification Kit, Qiagen, Germantown, MD, USA), sanger sequenced (Genewiz, Plainfield, NJ, USA), and cloned into the Gateway entry vector pCR8/GW/TOPO (Invitrogen, San Diego, CA, USA) according to the manufacturer’s instruction. Orientation was confirmed by sequencing the plasmids from selected colonies. Entry clones were sub-cloned into the BiFC plasmid sets pSAT5-DEST-cEYFP(175-end)-C1(pE3130), pSAT5(A)-DEST-cEYFP(175-end)-N1 (pE3132), pSAT4(A)-DESTnEYFP(1-174)-N1 (pE3134), and pSAT4-DEST-nEYFP(1-174)-C1 (pE3136) destination vectors (https://www.bio.purdue.edu/people/faculty/gelvin/nsf/protocols_vectors.htm, accessed on 16 June 2023) using Gateway LR clonase-II enzyme mix (Invitrogen, USA). Orientation was further confirmed by sanger sequencing of the fusion plasmids isolated (PureLink^®^ HiPure Plasmid Midiprep Kit, Invitrogen, USA) from selected colonies using primers listed in Supplementary Table S3. Each pair of recombinant plasmids encoding nEYFP and cEYFP fusions was mixed 1:1 (*w*/*w*), and co-bombarded with gold particles (1 um, Au, Bio-Rad) into onion epidermal layers. Each pair of recombinant plasmids encoding nEYFP or cEYFP fusion proteins was co-bombarded into onion epidermal cells using Helios DNA particle delivery system (Biolistic PDS-1000/He, Bio-Rad, CA, USA) as described by Hollender and Liu (2010) [96]. Bombarded epidermal cells were incubated in MS liquid media for 16–24 h at 22 °C under dark incubation, followed by observation for YFP fluorescence with an inverted spinning-disk confocal microscope (Eclipse Ti-E-PFS, Nikon, NY, USA). For salt treatment, onion cells were incubated in 200 mM NaCl for 30 min before imaging. Confocal fluorescent images and DIC images were acquired and processed using the Nikon NIS-Elements software. AtRACK1A was used as a positive control, as described by Sabila et al., 2016 [68]. Negative controls for interaction were provided by empty YFPC and YFPN vectors in combination with the OsRACK1B and OsSGR pSAT expression plasmids (Methods S2). Primers used for BiFC plasmids are listed in Supplementary Table S3.

### 4.6. Leaf Disc Assay and Chlorophyll Pigment Analysis

Leaf discs were excised from fully expanded leaves of eight-week-old WT and transgenic plants. Then, leaf strips were floated on water (as experimental control) or 200 mM NaCl (for salinity stress) in 6-well plates. Plates were kept under continuous light for 72 h at room temperature. After 72 h, leaf discs were photographed (Leica EZ4 stereo microscope) and used for spectrophotometric measurement of chlorophyll contents using protocols described previously [97]. Briefly, equal-size leaf discs were incubated with 7.5 mL DMSO at 65 °C for 30 min. The extracted chlorophyll solution was made up to 10 mL, with DMSO. A total of 3 mL of the total extracted solution for each sample was transferred to a cuvette, and the OD values were measured at 645 and 663 nm using GENESYS 20 UV/Vis spectrophotometer (Thermo Fisher Scientific, MA, USA). The chlorophyll concentration was calculated as described by Arnon (1949) [98] using the following equation:

Total Chlorophyll (μg/mL) = 0.0202 A663 + 0.00802 A645, where: A645 = absorbance at a wavelength of 645 nm; A663 = absorbance at a wavelength of 663 nm.

For pigment analysis, the acquired images were processed using the free ImageJ software (Rasband, WS, ImageJ, US National Institutes of Health, Bethesda, MD, USA). Color thresholding was applied in RGB color space, adjusting the parameters in order to select the green pigments and separate them from the background.

## 5. Conclusions

In this work, we reveal the physiological function of RACK1 in chlorophyll catabolism, which was enigmatic since, to date, reports have only indicated its interaction with photosynthesis-related proteins. Delaying senescence or increasing chlorophyll stability during stress conditions is linked to increased photosynthetic efficiency and improved grain yield [99,100]. Grain filling and increased yields are agronomic traits of interest. Therefore, our study provides important insights into the molecular mechanisms of salt-tolerance and could be used to ameliorate the effect of salt on photosynthesis to increase the yield potential of rice, an important cereal crop, under current global climate change.

## Data Availability

Not applicable.

## Supplementary Materials

The following supporting information can be downloaded at: https://www.mdpi.com/article/10.3390/plants12122385/s1, Figure S1: Identification of T-DNA insertion mutant of RACK1B (LOC_Os05g47890). Figure S2: Diagnostic PCR for duplication of T-DNA. Figure S3: Changes in total chlorophyll content in OsRACK1B transgenic rice leaves during salinity stress. Figure S4: BiFC analysis of the interaction between rice RACK1B and SGR in the Nucleus and Cytoplasm in onion epidermal cells under salinity stress. Table S1: Primers used for genotyping of T-DNA insertional mutagenesis lines. Table S2: Primers used for quantitative Real time PCR. Table S3: Primers used for BiFC assay and plasmid sequencing.
