# Supplementary material for: Receptor for Activated C Kinase1B (RACK1B) Delays Salinity-Induced Senescence in Rice Leaves by Regulating Chlorophyll Degradation
Source: Plants (Basel). 2023 Jun 20;12(12):2385. doi: 10.3390/plants12122385 (PMC10303231; doi:10.3390/plants12122385)
Supplement: Supplementary file 1 [file plants-12-02385-s001.zip › plants-2444070-supplementary.pdf]

Supporting Information

Article title: **Receptor for Activated C Kinase1B (RACK1B) Delays Salinity-induced Senescence in Rice Leaves by Regulating Chlorophyll Degradation**

Authors: Md Ahasanur Rahman, Hemayet Ullah

The following Supporting Information is available for this article:

Figure S1 Identification of T-DNA insertion mutant of RACK1B (LOC_Os05g47890)

Figure S2 Diagnostic PCR for duplication of T-DNA

Figure S3 Changes in total chlorophyll content in OsRACK1B transgenic rice leaves during salinity stress

Figure S4 BiFC analysis of the interaction between rice RACK1B and SGR in the Nucleus and Cytoplasm in onion epidermal cells under salinity stress

Table S1 Primers used for genotyping of T-DNA insertional mutagenesis lines

Table S2 Primers used for quantitative Real time PCR

Table S3 Primers used for BiFC assay and plasmid sequencing

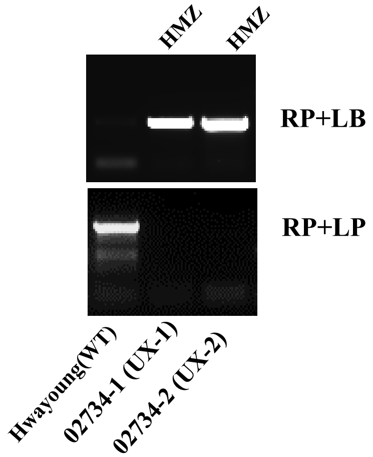

Figure S1: Identification of T-DNA insertion mutant of RACK1B (LOC_Os05g47890). Genotyping PCR of Salk line PFG_3D-027334 by T-DNA and gene-specific primers demonstrating homozygous (HMZ) T-DNA insertion in two PFG_3D-02734 lines (UX-1 and UX-2); Upper panel: T-DNA left border primer (LB) and a gene-specific primer (RP) amplified a ~500-bp PCR product from PFG_3D-027334 DNA (lane 2 and 3) but not from wild-type (WT) DNA (lane 1); Lower Panel: gene-specific primers (LP and RP) spanning the insertion site amplified a 1000-bp PCR product from wild-type DNA (lane 1) but not from two plants of PFG_3D-027334 DNA (lane 2 and 3) demonstrating *rack1b* homozygous (HMZ) T-DNA insertion in the PFG_3D-027334.

Genotyping PCR of RACK1B overexpressed Salk lines PFG_3A-07870 and PFG_3D-60781 by T-DNA and gene-specific primers can be found in Rahman et al., (2022).

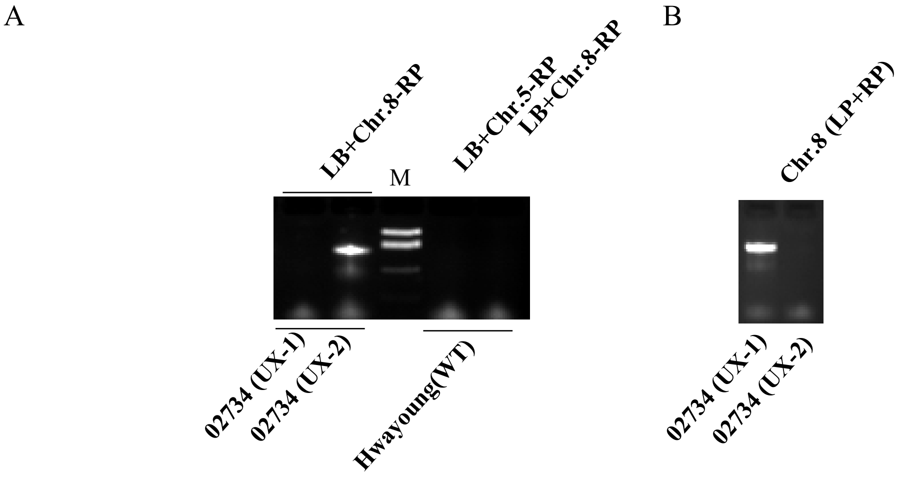

Figure S2: Diagnostic PCR for duplication of T-DNA. T-DNA insertion at LOC_Os08g0558200 in one of the two rice plants of line PFG_3D-02734. Genomic DNA from wild-type (WT) and *rack1b* homozygous mutant plants (PFG_3D-02734) was amplified using the primer combinations indicated. A. Combination of T-DNA left border primer (LB) and gene (LOC_Os08g0558200) specific right border

primer (Chr.8-RP) amplified a ~700 bp pcr product (lane 2) from UX-2 plant only but not from UX-1 (lane 1) and WT (lane 5). B, by using gene specific primers (RP and LP) from gene LOC_Os08g0558200, a ~1100 bp PCR product (lane 1) was amplified from PFG_3D-027342-1 (UX-1) but not from UX-2 (lane 2) showing the disruption of LOC_Os08g0558200 by T-DNA.

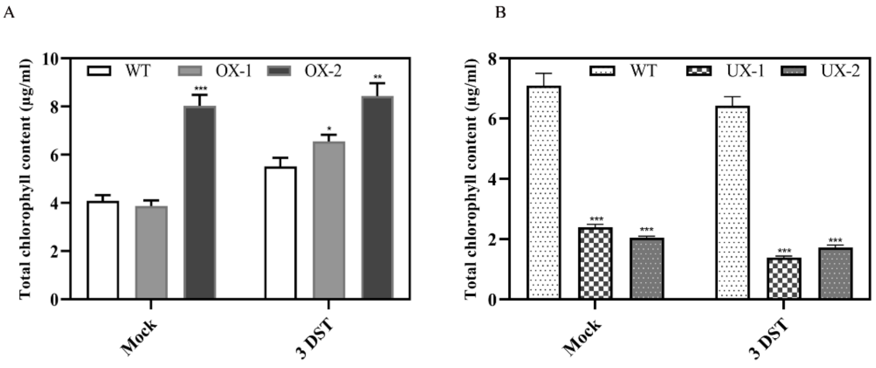

Figure S3: Changes in total chlorophyll content in OsRACK1B transgenic rice leaves during salinity stress. A. Retention of total chlorophyll content in leaf discs from OsRACK1B overexpressed rice plants OX-1 and OX-2 exhibiting the stay-green phenotype after 3 days of salt DST (200mM NaCl) treatment and 3 days of water treatment (Mock) as control in comparison to the WT (DnJ) leaf discs.

B. Reduced chlorophyll content in the OsRACK1B down-regulated rice plant UX-1 and UX-2 leaf discs exhibit yellowing (premature senescence) phenotype than wild-type (WT, Hwy) during 3 days of salt treatment (3 DST) and 3 days of water treatment (Mock). Each value represents the mean of three replicates ± SE. The asterisk indicates $p<0.05$, double asterisk indicates $p<0.01$, and triple asterisk indicates $p<0.001$, respectively (student's t-test) compared to Control groups. The experiments were repeated at least three times with similar results.

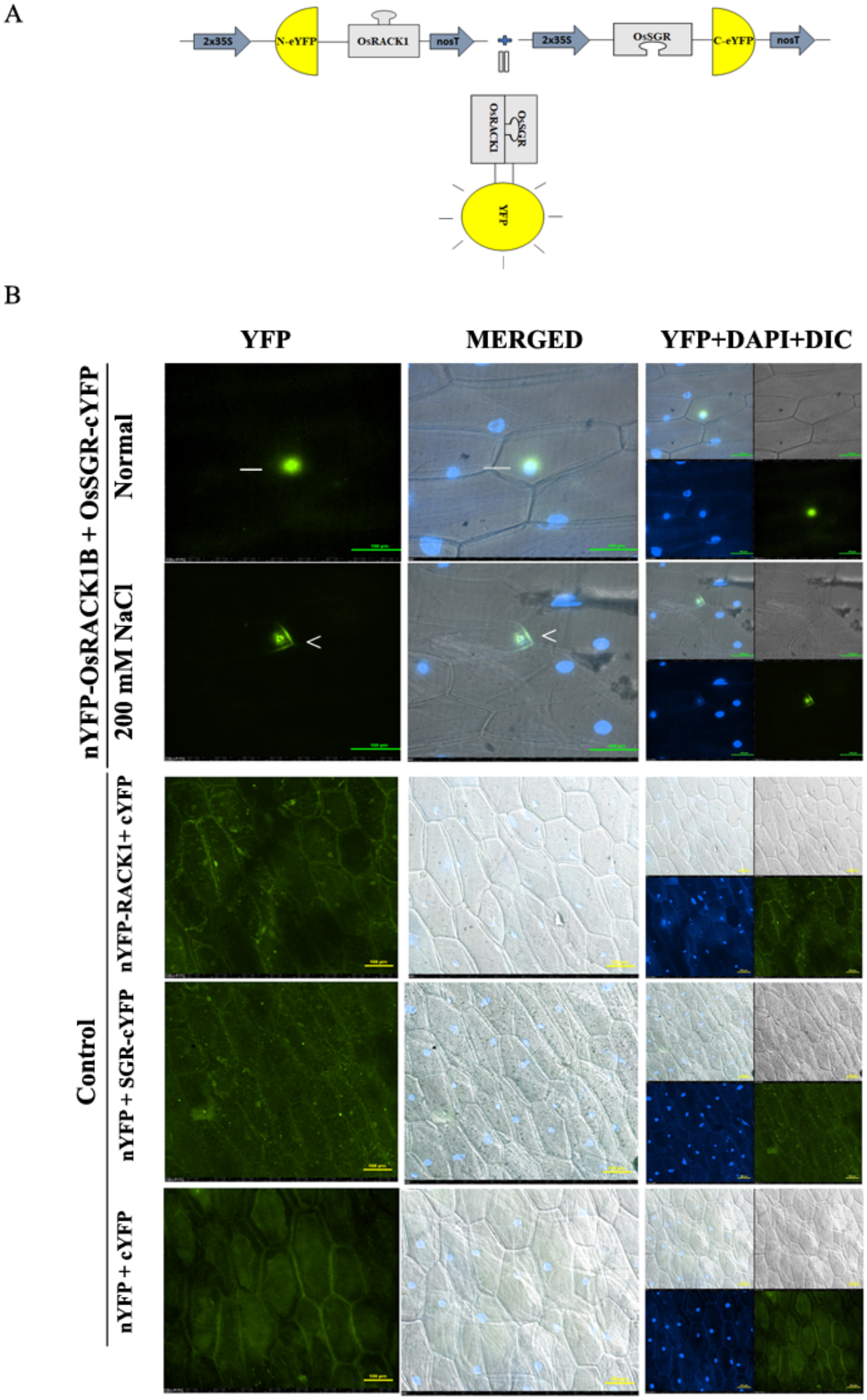

Figure S4: *In Vivo* interactions between RACK1 and SGR1 in onion epidermal cells analyzed by BiFC. **A**, A representative illustration of the constructs used in the BiFC assay. The Yellow Fluorescent Protein (YFP) is split into two nonfluorescent halves (YN and YC) which are fused to OsRACK1 and OsSGR. Specific protein-protein interaction results in a reconstructed Yellow Fluorescent Protein signal. **B,** The onion epidermal cells were transiently transformed with the full-

length OsRACK1B and OsSGR coding sequences. Straight line indicating green signals show the binding of nYFP-RACK1 with SGR-cYFP in the nuclei (normal) and arrow head shows the interaction in the cytoplasm (in salt stress condition). Blue fluorescence from DAPI (4 9, 6-diamidino-2-phenylindole) staining indicates nuclei, Merge, merged images of YFP channel, DAPI and differential interference contrast image (DIC). Constructs were fused to either carboxy (YFPC) or amino (YFPN) terminus of YFP and vice versa. All constructs were under the control of the 35S promoter. Each pair of recombinant plasmids encoding nYFP and cYFP fusions was mixed 1:1 (w/w) and co-bombarded into onion epidermal cell layers. The transformed onion epidermal layers were incubated at 22 °C for 16–24 h under dark condition. For salt treatment, epidermal cells were incubated in 200 mM NaCl for 30 minutes before imaging. The coexpression of nYFP with cYFP, nYFP with SGR-cYFP, and nYFP-RACK1 with cYFP serve as negative controls as there are no YFP signals observed.
Bars = 100 μm.

Table S1: Primers used for genotyping of T-DNA insertional mutagenesis lines

Primers	Insertion Chromosome	Sequence (5′→3′)	Purpose
LP	Chr05	TGCTCAATCAAAAGGGGTATG	Genotyping of Salk line: PFG_3D-02734.L
RP		AAGGTCTGGAACCTCACGAAC	
LB+115	T-DNA left border	GATCCGAAACTATCAGTGTCT	
LP	Chr08	TGTGTTTTTCACGTGCACAC	Genotyping of Salk line: PFG_3D-02734.L
RP		TGGTGACTAGCGAGTTGTGC	
F99	T-DNA left border	TTGAGACTTTTCAACAAAGG	

Table S2: Primers used for quantitative Real time PCR

Gene	Accession no.	Primer Sequence (5′→3′)	Product Size (bp)
OsRACK1B	Os05g47890	Forward-ATGGCGGGCCAGGAGTCGCTCACC<br>Reverse-TCCCAGGATCCCGAGAGCGCGAACT	290
OsCAO	Os10g41780	Forward-ACAAAACCACCCTCGGTTGA<br>Reverse-CCAACACCCTTTCTGGAGCA	173
OsSGR	Os09g0532000	Forward-CTGCAGGGGTGGTACAACAA<br>Reverse-TGGACGAACGCCTTCAGAAC	187
OsRCCR1	Os10g038920	Forward-AGCACCTTCTCACTGACAGC<br>Reverse-TGAAGAAGTGCCCTAGCAGC	116

OsNYC1	Os01g0227100	Forward-CACTTGCTCGGGAGTTCCTT<br>Reverse-CAACTGACAAGCCCTCCTGT	120
OsSTN7	Os05g0549100	Forward-AACGGACAGCAGCCTCATAC<br>Reverse-AAGATGTCAAAGCCCCTCCG	123
OsNAC092/ ORE1	Os04g046060	Forward-GACGCTCGTTTTCTACACGG<br>Reverse-TGCACAACACCCACTCGTT	152
OsActin1	Os03g50885	Forward-TCCATCTTGGCATCTCTCAG<br>Reverse-TGGCTTAGCATTCTTGGGTC	126

Table S3: Primers used for BiFC assay and plasmid sequencing

Gene/Primer	Accession/Vector	Primer sequence (5′→3′)	Purpose
OsSGR	Os09g0532000	Forward-<br>ATGGCTGCTGCTACTTCGACCATGTC<br>Rev w/o stop-<br>CTGCTGCGGCTGGCCGTCGGC<br>Rev with Stop-<br>TCACTGCTGCGGCTGGCCGT	Full length CDS amplification
OsRACK1B	Os05g47890	Forward- ATGGCGGGCCAGGAGTCG<br>Rev with stop-<br>CTAGATTGCATAGCCGCCAAACC<br>Rev w/o stop-<br>GATTGCATAGCCGCCAAACCCT	Full length CDS amplification
OsRACK1B -445		Forward-GGCGAGTGCAAGTACACCAT	Sanger Sequencing
OsRACK1B -860		Forward-ACCTCAAGCCAGAAGTCCAG	Sanger sequencing
GW1	TOPO TA cloning vector	GTTGCAACAAATTGATGAGCAATGC	Sequencing the insert
GW2		GTTGCAACAAATTGATGAGCAATTA	
EGFP-N	Destination Vector	CGTCGCCGTCCAGCTCGACCA	Sequencing OsRACK1B and OsSGR-n/c-YFP vectors
EGFP-C-FOR		CATGGTCCTGCTGGAGTTCGTG	
Forward1		GAGCTGAAGGGCATCGACTT	
Reverse2		TTGTACAGCTCGTCCATGCC	
